# Accurate measurement of liquid transport through nanoscale conduits

**DOI:** 10.1038/srep24936

**Published:** 2016-04-26

**Authors:** Mohammad Amin Alibakhshi, Quan Xie, Yinxiao Li, Chuanhua Duan

**Affiliations:** 1 Department of Mechanical Engineering, Boston University, 110 Cummington Mall, Boston, MA, 02215, USA

## Abstract

Nanoscale liquid transport governs the behaviour of a wide range of nanofluidic systems, yet remains poorly characterized and understood due to the enormous hydraulic resistance associated with the nanoconfinement and the resulting minuscule flow rates in such systems. To overcome this problem, here we present a new measurement technique based on capillary flow and a novel hybrid nanochannel design and use it to measure water transport through single 2-D hydrophilic silica nanochannels with heights down to 7 nm. Our results show that silica nanochannels exhibit increased mass flow resistance compared to the classical hydrodynamics prediction. This difference increases with decreasing channel height and reaches 45% in the case of 7 nm nanochannels. This resistance increase is attributed to the formation of a 7-angstrom-thick stagnant hydration layer on the hydrophilic surfaces. By avoiding use of any pressure and flow sensors or any theoretical estimations the hybrid nanochannel scheme enables facile and precise flow measurement through single nanochannels, nanotubes, or nanoporous media and opens the prospect for accurate characterization of both hydrophilic and hydrophobic nanofluidic systems.

Understanding liquid transport through nanoscale confinements is critical in a variety of practical applications, including energy conversion/storage^1^,^2^, water desalination^1^,^3^, phase-change thermal management^4^, biological and chemical separations^5^, and lab-on-a chip devices^6^. Although it has been argued that continuum assumption and classical hydrodynamics are capable of describing liquid transport at the nanoscale^1^,^7^,^8^,^9^, the differences between nanoscale and micro/macroscale liquid transport, in terms of confined liquid properties^9^,^10^,^11^,^12^,^13^,^14^,^15^,^16^,^17^,^18^, flow boundary conditions (slip/no slip)^9^,^18^,^19^,^20^,^21^,^22^,^23^, secondary flows^24^,^25^,^26^, etc, still remain elusive. In fact, a wide range of slip length and confined liquid properties with up to several orders of magnitude discrepancies between different sources have been reported, indicating that nanoscale liquid transport has remained poorly characterized and novel accurate measurement techniques for this purpose are desired. The major challenge in performing precise flow measurement in nanoscale conduits is the associated huge hydraulic resistances which result in ultra-small flow rates. For example, based on classical hydrodynamics only ~0.25 al/s water flows through a hydrophilic tube 100 micron long and 10 nm in diameter, when one atmosphere pressure is applied. The most common method to bypass this challenge is to measure liquid transport in membranes consisting of numerous similar nanoscale conduits^19^,^20^,^21^,^22^. However, analysis and verification of the data from this method is complicated by the fact that the measured flow rate constitutes an average over a large unknown number of conduits with a range of diameters and lengths. There are also concerns about possible leakage due to membrane defects and/or leakage at the membrane’s edges^27^. Moreover, it may not be possible to create membrane structures for certain nanoscale conduits with specific geometry and surface properties, e.g. 2-D planar nanochannels that are widely used in lab-on-a-chip devices. On the other hand, capillary flow measurement is the major method used for characterizing fluid flow in individual nanoscale conduits by tracking the location of a moving meniscus as a function of time^18^,^28^,^29^,^30^,^31^,^32^,^33^,^34^,^35^,^36^. However, in this method–which is mainly applicable to hydrophilic channels–the driving pressure in the nano-conduits is not experimentally measured, but calculated based on classical theories^28^,^29^,^30^,^31^,^32^,^33^,^34^,^35^,^36^ with bulk liquid properties or molecular simulations^18^, which can be quite different from the actual values, resulting in inaccurate calculation of the actual hydraulic resistance. Given the limitations of the current measurement techniques, it is thus necessary to develop a technique for liquid flow measurement in single nano-conduits^37^,^38^ which can be applied to both hydrophobic and hydrophilic conduits without using any theoretical estimations. Herein, we report such a technique based on capillary flow and a novel hybrid nanochannel design and use it to characterize water transport in single silica nanochannels with heights down to 7 nm.

## Hybrid Nanochannel Scheme

**Figure 1:**
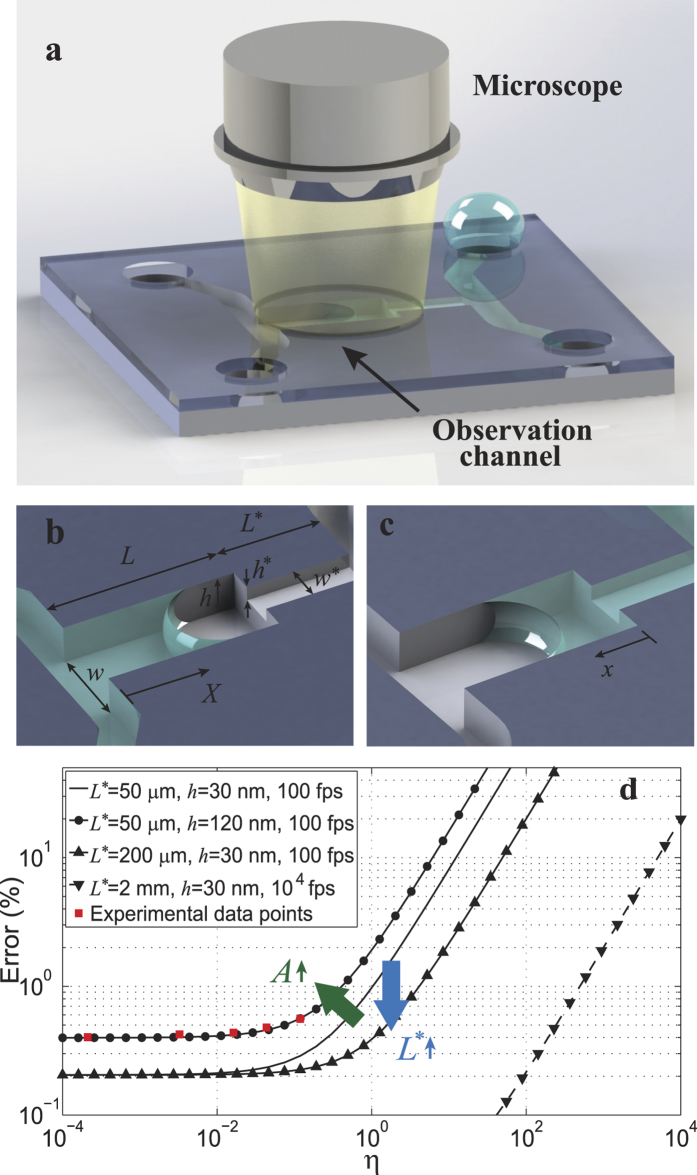
Schematic of the hybrid nanochannel scheme for nanoscale liquid transport characterization and error analysis. (**a**) Channel under investigation is seamlessly connected to a reference channel of known hydraulic resistance, and the data is collected from the observation channel which can be either of the channels. (**b**) The Observation channel is first characterized by a capillary flow experiment yielding the value of *A* which is a function of both hydraulic resistance and the capillary pressure only in this channel. (**c**) Ratio of the mass flow resistance between the two channels (*η*) can be found by introducing liquid from the other side, and tracking the meniscus again in the observation channel. (**d**) Error associated with *η* other than the temporal and spatial dependence is a function of *A* and L*. Assuming *δx* = *δX* = 1 *μ*m, and the observation channel is 350 *μ*m long, error for different values of A, L*, and frame rates has been evaluated. (The square markers are the experimental data points).

The hybrid nanochannel design consists of a test channel (the channel under investigation) seamlessly connected to a reference channel with a different but known mass flow resistance (Fig. 1). In a typical experiment, two capillary flow measurements are conducted in the hybrid channel, one starting from the test channel side and the other starting from the reference channel side. However, the meniscus location is only recorded in one of the two channels, which we call it the “observation channel”. Without loss of generality, let’s assume the observation channel is the reference channel. In this case, the first capillary filling process starts from the reference channel side (Fig. 1b). The location of the meniscus in the reference channel *X*(*t*) is recorded and is expected to be governed by the Washburn equation, which for the case of a channel with rectangular cross-section can be written as (Supplementary information, section I)


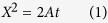



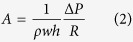


here, Δ*P* is the capillary pressure, *R* is the mass flow resistance of the channel per unit length, *w* and *h* are channel width and height, and *ρ* is the fluid density. After the first measurement, liquid is removed from the nanochannel and is introduced from the test channel side for the second measurement (Fig. 1c). The location of the meniscus in the reference channel during the second capillary filling process *x*(*t*) is then recorded, which is expected to be described by the following equation (Supplementary information, section I):


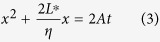



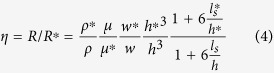


In this equation, *t* = 0 corresponds to the time when the meniscus enters the reference channel (*x* = 0, Fig. 1c), *η* is the ratio of the mass flow resistance between the reference channel and the test channel per unit length, *μ* is the fluid viscosity, *l*_*s*_ is the slip length, and the starred variables belong to the test channel, with L* being the length of the test channel. By fitting experimental data sets *X*(*t*) and *x*(*t*) with equations (1) and (3), capillary flow constant, *A*, in the observation channel and the mass flow resistance ratio, *η*, can be extracted. Consequently, resistance of the test channel can be quantified if the resistance of the reference channel is known. It is worth noting that the test channel can be an individual channel, tube or even nanoporous media, and no matter if it is hydrophobic or hydrophilic, as long as either of test or reference channels allows for spontaneous liquid filling, this technique can be applied. (Supplementary information, section IV.C).

The hybrid channel scheme can be used to measure a wide range of *η*’s and thus a wide range of nanochannel resistance, with a relatively small error. Our linear regression based error analysis indicates that the experimental error associated with *η* (*E* = *δη*/*η*) reaches a plateau at small values of *η* determined by 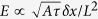, with *δx* and *τ* being the spatial resolution and the frame interval (Fig. 1d, Supplementary information, section II). In this range, error is very small and is not a function of *η* and L*. At large values of *η*, however, error is a linear function of *η* (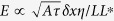), and a larger L* along with a smaller capillary flow constant *A* can be employed to reduce the error in this range. In fact, this method can be best utilized if *η* is smaller than 1. To study test channels with a very small resistance compared with the reference channel (yielding large values of *η*), one should possibly choose the test channel as the observation channel such that 1/*η* would be measured instead of *η*. This resolves the theoretical limit for accurate characterization of the flow and allows for measurement of very high resistance ratios with a small error. Nevertheless, by designing a long test channel and choosing a high frame rate, the hybrid nanochannel scheme is able to detect very large values of *η* (>10^4^) with a small error (Fig. 1d). Therefore, this scheme is adequate for the study of enhanced liquid transport in carbon nanotubes and graphene nanochannels where a wide range of flow enhancements have been reported^9^,^18^,^19^,^20^,^21^.

## Design, Fabrication, and Measurement

**Figure 2:**
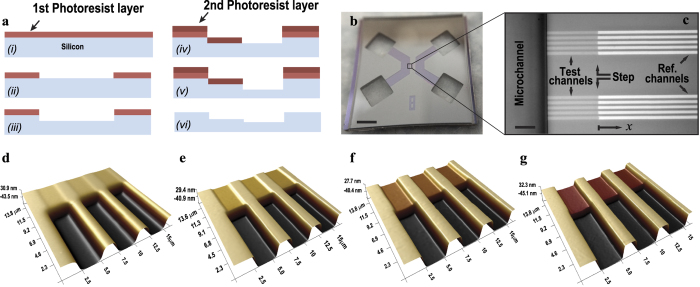
Device structure and characterization. (**a**) Fabrication procedure of the hybrid channels, using double layer photoresist coating and RIE etching. (**b,c**) structure of a chip used for experiments; water is carried from one of the reservoirs to the nanochannels through a microchannel. Nanochannels 600 micron long are located between two microchannels, each consisting of a shallow side and a deep side creating a step in the nanochannels. (scale bars of (**b**,**c**) are 2 mm and 20 micron) (**d–g**) Representative AFM images of four hybrid channels used for the experiments with h*/*h* = 7 nm/117.5 nm, 16.2 nm/109.5 nm, 38 nm/108 nm, and 59 nm/121 nm.

In the present investigation, the proposed characterization scheme is utilized to study water transport in hydrophilic silica nanochannels with heights ranging from 7 nm to 59 nm. In our design, nanochannels with the same widths but larger heights (~110–120 nm) serve as the reference channels. This choice of the reference channel height serves several purposes: First, this depth of water in the reference channel can be very easily detected with an optical microscope. Second, this choice of height helps to avoid entrapment of air and creation of bubbles in the reference channel^33^,^34^,^35^,^36^. Finally, with this choice of heights, *η* would be less than 1 and the experimental error would be very small (Fig. 1d). The hybrid silica nanochannel devices are fabricated using the classic etching and bonding scheme, while the seamless connection between the test and the reference channels are achieved using double-layer photoresist coating (Fig. 2a). Briefly, five sets of stepped nanochannels were fabricated with h*/*h* = 7 ± 0.5 nm/117.5 ± 0.5 nm, 16.2 ± 0.4 nm/109.5 ± 1 nm, 28 ± 0.5 nm/110.5 ± 2 nm, 38 ± 0.5 nm/108 ± 1 nm, and 59 ± 0.5 nm/121 ± 1 nm. Uniformity of the RIE etching throughout the entire silicon wafers allowed us to fabricate many chips of almost exact heights in each trial. The width of both test and reference channels is 3 *μ*m, the reference channel’s length is *L* = 550 *μ*m, and the test channel’s length is L* = 50 *μ*m, except for the 7 nm channel which is L* = 7.5 *μ*m. Long test channels with very small heights impose a very large resistance before the reference channel such that the meniscus may stop at the step and the corner flows become the dominant mode of filling^39^,^40^,^41^ (Supplementary Figure S5). For this reason the 7 nm test channel is chosen to be 7.5 micron long to allow easy flow of water. After etching the nanochannels, two microchannels each 6 mm long, 1 mm wide and 40 *μ*m deep were etched using DRIE on both terminals of the nanochannels to carry water from the reservoirs, and the four reservoirs which are 2 mm by 2 mm through holes were later etched using DRIE (Fig. 2b). Finally, 300 nm thick dry thermal oxide layer was grown on the silicon chips, and the chips were cleaned with Piranha (3:1, *H*_2_*SO*_4_:*H*_2_*O*_2_) and bonded to Borosilicate glass by using anodic bonding at 400 °C and 350 Volts. Miscroscope images of a bonded hybrid nanochannel device is shown in Fig. 2b. The test channels, reference channels, location of the steps, as well as connection of the microchannel to the nanochannels for this device (16.2 nm/109.5 nm) is shown in Fig. 2c. Heights of the test and the reference nanochannels are measured using AFM before anodic bonding. Figure 2d–g show the 3D AFM images of four representative hybrid nanochannels used for the experiments with h* = 7, 16.2, 38, and 59 nm.

All the experiments were performed with DI water (electrical resistivity >18 MΩ-cm) at 22 ± 1 °C (*pH* = 7), before each experiment oxygen plasma was applied to the chips for 15 minutes to make the surfaces super hydrophilic and prevent formation of bubbles in the nanochannels. The water Meniscus in nanochannels were tracked by an Olympus inverted microscope model *IX*81 equipped with a monochromatic HAMAMATSU CMOS FLASH 4.0 C11440 camera recording at up to 900 fps (or at lower rates when not necessary). Position of the meniscus as a function of time was extracted from the recorded frames using a MATLAB image processing code.

## Results and Discussion

**Figure 3:**
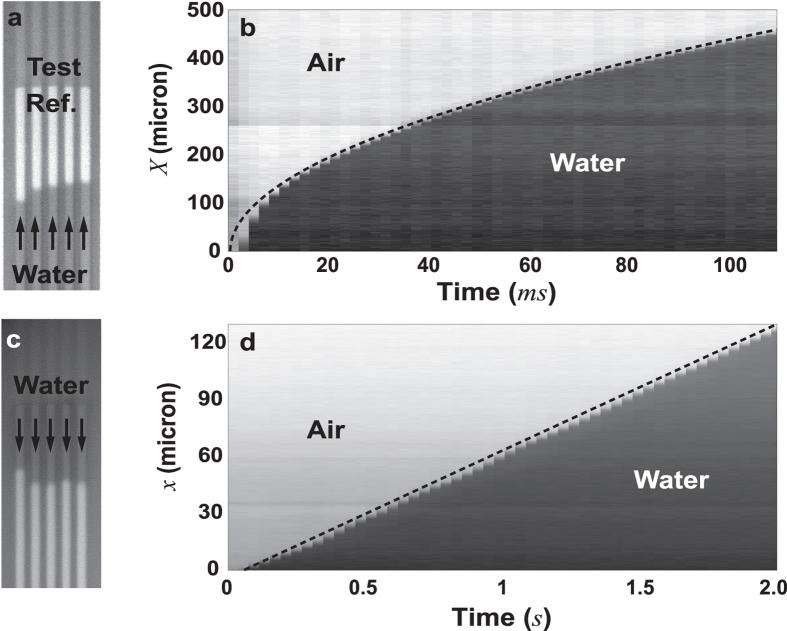
Capillary filling of a hybrid nanochannel. (**a**) Microscope image of capillary filling of the reference channel starting from the reference channel side. Image processing of the recorded frames allows for extraction of *X* − *t* curve of the meniscus. (**b**) Meniscus location in the reference channel recorded at 500 fps, showing a clear square root time dependence. (**c,d**) Capillary filling of the reference channel starting from the test side (h* = 16.2 nm). Meniscus moves with a constant speed and the filling rate is 2 orders of magnitude slower than the first experiment.

The capillary flow constant, *A*, is found by introducing water from the reference channel side, tracking the location of meniscus as a function of time and curve fitting to the experimental data (Fig. 3a,b). This quantity is known to be smaller than theoretical predictions for the nanochannels and different reasons have been proposed to explain the discrepancies between theory and experiments^24^,^25^,^26^,^28^,^29^,^30^,^31^,^32^,^34^,^35^. (Supplementary information, section IV.A) Here, for the reference channels of similar height (110 nm to 120 nm), the measured correction factors defined as *C* = *A*_*theory*_/*A*_*actual*_ ranged from 1 to 1.35 with the average of *C* = 1.22. Moreover, we observed that this correction factor increases over time, which is mainly attributed to deterioration of surface properties and creation of hydrophobic sites along the channels. (Supplementary information, section IV.B) After measuring the capillary flow constant, in the second experiment water is introduced from the test channel side. (Figure 3c,d, h* = 16.2 nm) It was observed that meniscus moves with a constant speed (linear dependence of *x* on *t*, instead of 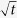), consistent with equation (3) in case of *η* ≪ 1:


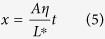


In fact, when *η* ≪ 1, most of the resistance originates from the test channels and as a result, Δ*P* at the meniscus has to overcome an almost constant resistance throughout the entire filling process and thus travels with a constant velocity. This velocity is much slower than the first experiment which allows for easy measurement of the *x* − *t* data at low frame rates.

**Figure 4:**
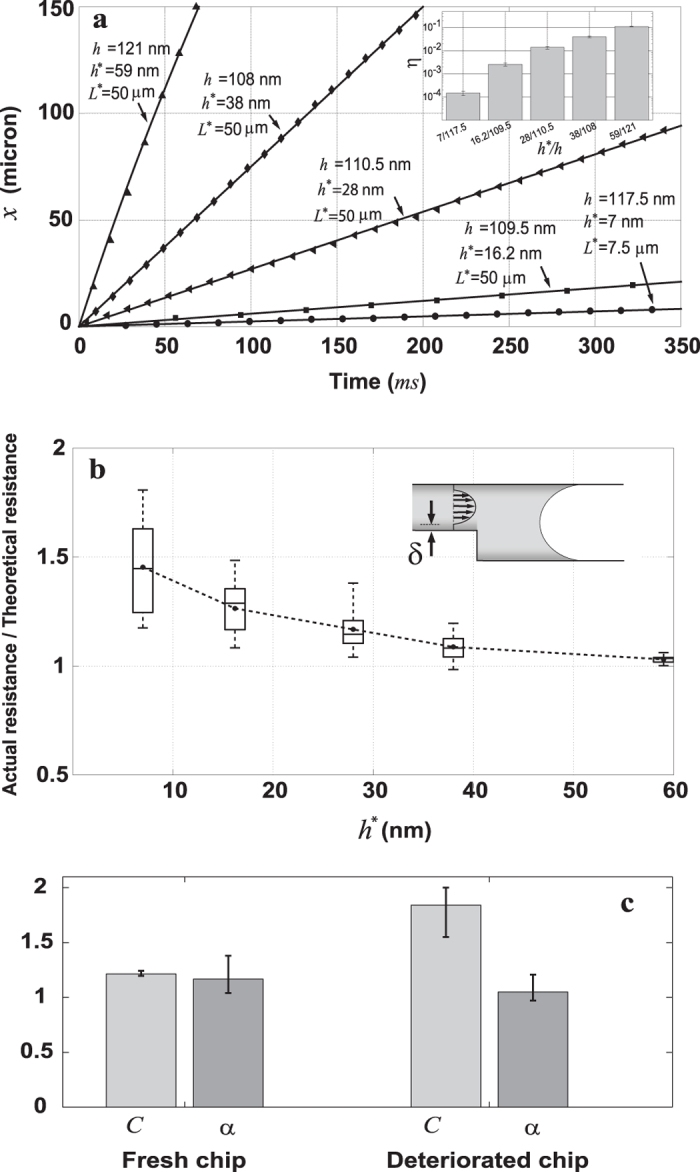
Location of the meniscus versus time, *α* versus channel height, and decoupling of *α* from *C*. (**a**) Representative curves of the location of meniscus versus time for hybrid nanochannels with different *η*’s. Solid lines are fitted curves to the experimental results (markers). For *η* ≪ 1, the linear relation between *x* and *t* signifies the hydraulic resistance is almost entirely caused by the shallow test nanochannels. (**b**) Ratio of the actual resistance of the nanochannels to the theoretical resistance (*α*) vs nanochannel height. Formation of a stagnant layer of water on the silica surface with the thickness of *δ* = 4.3, 7.1, 8.9, 8, and 7 angstroms (from shallow to deep) can explain the increased resistance. (**c**) Characterizing the increased hydraulic resistance for a 28 nm channel measured with two chips of different hydrophilicity. Decrease in the capillary pressure due to change in hydrophilicity of the surfaces does not impact the measured hydraulic resistance.

Representative measured *x* − *t* curves for each of the five tested hybrid nanochannel sets along with the experimental *η*’s are presented in Fig. 4a. Our experimental results measure *η*’s spanning over three orders of magnitude with a small error predicted by our error analysis (Figs 1d and 4a). Theoretical values of *η* normalized by the experimental *η* yield the increase in the resistance as a function of height: *α* = *η*_*theoretical*_/*η*_*actual*_ = *R*_*test*,*actual*_/*R*_*test*,*theoretical*_. An implicit assumption here is that resistance of ~120 nm reference channels can be approximated by the classical equation for *R*, otherwise a slight increase in the measured *α* can be expected. Our results indicate that for the 59 nm channels the actual resistance is very close to the theoretical prediction and as the test channel height becomes smaller, difference between the actual and the theoretical resistance becomes more pronounced, with the ratio reaching *α* = 1.45 ± 0.31 in case of 7 nm channels (Fig. 4b). As it’s clear from the definition of *R*, within the realm of hydrodynamics the liquid-surface interaction can manifest itself in the boundary conditions, i.e., slip/no-slip boundary condition, and/or in the form of an altered liquid property (i.e., density and viscosity). In terms of the boundary condition, slip boundary condition has been reported for the hydrophilic channels^22^,^23^. However, any non-zero slip lengths could only reduce the resistance and hence can not serve as an explanation for our results (here *R*_*theoretical*_ is calculated with *l*_*s*_ = 0). In terms of liquid properties, several of previous studies have employed long-range electrostatic forces to explain the slow capillary filling in hydrophilic nanochannels through the Debye-layer correction for the hydraulic resistance. However, this effect known as electroviscosity has been proven insignificant for realistic estimates of central parameters^24^,^25^,^26^. On the other hand, change in the interfacial liquid properties due to short-range interaction forces can be another way to explain the increased resistance^32^,^37^. When water is in contact with a polar surface, water dipoles reorient making formation of stagnant/tenacious hydration layers at the interface favorable. Experimental studies concerning fluidity of confined water between hydrophilic surfaces less than a few nanometers apart have demonstrated 1 to 6 orders of magnitude increase in water viscosity due to ordering of the water structure at the hydrophilic surfaces^10^,^11^,^12^,^13^,^14^, although there are other studies indicating this increase is not more than three-folds^15^,^16^,^17^. Moreover, MD simulations indicate a three to five fold increase in water density near hydrophilic surfaces^10^,^15^,^17^. Such changes in interfacial liquid properties, or formation of an ice-like network of water adsorbed on silica^42^, can reduce the effective flow cross-sectional area and hence be a possible explanation for our measurements. According to our measurements, a stagnant layer of water on the silica surfaces with thickness of 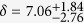 angstroms corresponding to two to three layers of water molecules can explain the observed increase in the mass flow resistance. However, since the fabrication method we have used involves anodic bonding, there is a concern about decrease in channel height after bonding. (Supplementary information, section III) Of course if any reduction in the channel height is going to happen after bonding, it must be subtracted from the measured thickness of the hydration layer. When comparing our results with previously reported hydration layer thickness of 5 angstroms^43^ or 4 angstroms^37^ in capillary filling studies one may argue that heights of our channels might have decreased by a few angstroms after bonding. However, it is worth noting that our measured value is in agreement with infrared reflection spectroscopy of water adsorbed on hydrophilic silicon oxide surfaces, which revealed that the first three adsorbed water layers (~8.4 angstroms thick) have an ice-like configuration^42^. Haneveld *et al*. also reported a thickness of *δ* = 9 ± 5 angstroms for the hydration layer in sub-10 nm nanochannels in their capillary filling study^31^, although their results may have overestimated the thickness of the hydration layer as they attribute deviations of both capillary pressure and hydraulic resistance from theory to the increase in hydraulic resistance. Additional possible sources of error for such measurements include capillary pressure induced channel wall deflection and dissolution of silica in water over time^44^. Nevertheless, the capillarity-induced channel wall deflection during water filling^32^,^45^,^46^,^47^ can be safely ignored for our nanochannels because of the thick cover layers (i.e., 0.5 mm thick glass/silicon). Moreover, the effect of channel wall dissolution (dissolution rate ~45 pm/hr^44^), a phenomenon observed when water flows in nanochannels over a long period, e.g. 48 hrs, is negligible as it only takes 1~2 minutes to perform multiple experiments in each nanochannel.

The hybrid channel design decouples two unknown parameters in conventional capillary flow measurements, i.e., driving capillary pressure and hydraulic resistance, holding promise to be the standard for nanofluidic flow characterization. The pressure-resistance decoupling in this method is further explored by testing hybrid channels with deteriorated surface properties which demonstrated correction factors *C* of up to 2 in the reference channels (Supplementary information, Figure S4a). In the deteriorated channels, while *C* was measured to be ~50% larger than a fresh chip, *α* maintained its value within 10% of the fresh chips, verifying that hybrid nanochannel scheme can avoid a large error arising from any changes in the pressure term (Fig. 4c).

In summary, our experimental results characterize water transport in sub-10 nm hydrophilic nanochannels and lends more validity to the use of classical hydrodynamics at the nanoscale. The proposed hybrid nanochannel scheme provides insight into collective effects of the boundary condition as well as the properties of a nanoscale confined liquid, and can open the prospect for accurate characterization of liquid transport through 2-D nanochannels, 1-D nanotubes, as well as nanoporous media. In particular, characterization of water transport in hydrophobics CNTs and graphene nanochannels can be improved through their integration with hydrophilic channels in the form of hydrophobic-hydrophilic hybrid channels. This method has the potential to be standard for nanofluidic flow characterization and can serve to advance studies of many nanofluidics-involved disciplines, including membrane separation, soil science, colloid chemistry, biology and physiology.

## Additional Information

**How to cite this article**: Alibakhshi, M. A. *et al*. Accurate measurement of liquid transport through nanoscale conduits. *Sci. Rep.* **6**, 24936; doi: 10.1038/srep24936 (2016).

## Supplementary Material

Supplementary Information
