# Supplementary material for: Accurate measurement of liquid transport through nanoscale conduits
Source: Sci Rep. 2016 Apr 26;6:24936. doi: 10.1038/srep24936 (PMC4844961; doi:10.1038/srep24936)
Supplement: Supplementary Information [file srep24936-s1.pdf]

Accurate measurement of liquid transport through nanoscale conduits

Mohammad Amin Alibakhshi, Quan Xie, Yinxiao Li & Chuanhua Duan[†]

Department of Mechanical Engineering, Boston University, 110 Cummington Mall, Boston, MA, 02215, USA. [†]e-mail: duan@bu.edu

I. Mass flow resistance and Washburn equation

Take a rectangular-shaped channel, with width and height of w and h. If the width of the channel is much larger than its height ($w \gg h$), with the neglect of inertia term the momentum equation governing the incompressible flow in this channel can be written as (Figure 1b):

$$\mu \frac{d^2u}{dz^2} = \frac{dp}{dX} \tag{1}$$

with μ being the fluid viscosity and z the direction along the channel height. The slip boundary condition for the lower and upper walls of the channel ($z = 0, h$) can be expressed as:

$$\frac{u}{l_s} = \pm \frac{du}{dz}\bigg|_{z=0,h} \tag{2}$$

in which l_s is the slip length. Accordingly, the velocity profile and the mass flow rate can be found as:

$$u = \frac{1}{2\mu}\frac{dp}{dX}(z^2 - hz - hl_s) \tag{3}$$

$$\dot{m} = \rho w \int_0^h u dz = -\frac{\rho w h^3}{12\mu}\frac{dp}{dX}(1 + 6\frac{l_s}{h}) \tag{4}$$

wherein ρ is the fluid density. The slip boundary condition covers the "no slip" boundary condition if one simply sets $l_s = 0$. Now, defining hydraulic resistance as $r = \Delta P/\dot{m}$, the hydraulic resistance of a channel per unit length would be obtained as:

$$R = \frac{12\mu}{\rho w h^3(1 + 6\frac{l_s}{h})} \tag{5}$$

Combining equations (4) and (5) yields a relation for liquid meniscus location in the channel (X) as a function of time, when a pressure difference of ΔP is applied:

$$\begin{gathered} \rho w h \frac{dX}{dt} = \frac{\Delta P}{RX} \\ X^2 = 2At, \quad A = \frac{1}{\rho w h}\frac{\Delta P}{R} \end{gathered} \tag{6}$$

If the pressure difference is replaced by the capillary pressure, $\Delta P = \frac{2\sigma\cos(\theta)}{h}$, with σ being the surface tension and θ the contact angle, and assuming $l_s = 0$, the following equation, known as the Washburn equation, governing the location of a meniscus as a function of time in capillary fillings can be derived:

$$X = \sqrt{\frac{\sigma h \cos(\theta)}{3\mu}t} \tag{7}$$

One can use this resistance concept to derive a governing equation describing capillary flow in a hybrid channel (Figures 1b,c). If R^* is the resistance per unit length of the test channel with length L^*, and the meniscus is at location x in the reference channel, then

mass flow rate in the hybrid channel can be written as:

$$\dot{m} = \rho w h \frac{dx}{dt} = \frac{\Delta P}{Rx + R^* L^*}$$
$$(Rx + R^* L^*)dx = \frac{\Delta P}{\rho w h} dt$$
$$\frac{1}{2}x^2 + \frac{R^*}{R} L^* x = \frac{1}{\rho w h}\frac{\Delta P}{R} t$$

Defining $\eta = R/R^*$, this equation can be written as:

$$x^2 + \frac{2L^*}{\eta} x = 2At \tag{8}$$

II. Error analysis

Possible detection range of this method along with the expected error can be understood from a comprehensive error analysis. Without loss of generality let's assume the observation channel is the reference channel. Experimental A can be determined from a set of (T_i, X_i), $i = 1..m$, measured in the reference channel when the water is introduce from the reference channel side (Figure 1b), by minimizing error using the following relations:

$$\epsilon^2 = \sum_{i=1}^{m} (X_i^2 - 2AT_i)^2$$
$$\frac{\partial \epsilon^2}{\partial A} = 0$$
$$A = \frac{\sum_{i=1}^{m} X_i^2 T_i}{2\sum_{i=1}^{m} T_i^2} \tag{9}$$

Similarly, η can be found from a set of (t_i, x_i), $i = 1..n$, measured in the hybrid channel when the water is introduced from the test channel side (Figure 1c):

$$\epsilon^2 = \sum_{i=1}^{n} (x_i^2 + \frac{2L^*}{\eta} x_i - 2At_i)^2$$
$$\frac{\partial \epsilon^2}{\partial \eta} = 0$$
$$\frac{1}{\eta} = \frac{A}{L^*} \frac{\sum_{i=1}^{n} x_i t_i}{\sum_{i=1}^{n} x_i^2} - \frac{1}{2L^*} \frac{\sum_{i=1}^{n} x_i^3}{\sum_{i=1}^{n} x_i^2} \tag{10}$$

Given $\eta = f(t_i, x_i, A)$, the temporal error (E_t), the spatial error (E_x), and the error associated with A (E_A) determine the total error as:

$$E = \sqrt{E_A^2 + E_x^2 + E_t^2} \tag{11}$$

Calculations showed that the temporal error is insignificant compared to the other terms $(E_t \ll E_A, E_x)$, and can be safely ignored. Therefore, for the sake of brevity only derivations of E_A and E_x are presented.

Error associated with A: E_A can be expressed as:

$$E_A = \frac{1}{\eta} \left| \frac{\partial \eta}{\partial A} \delta A \right| \tag{12}$$

in which both $\frac{\partial \eta}{\partial A}$ and δA must be determined. δA can be written as:

$$\delta A = \sqrt{\sum_{i=1}^{m} (\frac{\partial A}{\partial X_i} \delta X)^2 + (\frac{\partial A}{\partial T_i} \delta T)^2}$$

From equation (9) we get $\frac{\partial A}{\partial X_i} = \sqrt{2A}\frac{T_i^{3/2}}{\sum\limits_{j=1}^{m} T_j^2}$. Given the very small contribution of the temporal error ($|\frac{\partial A}{\partial T_i}\delta T| \ll |\frac{\partial A}{\partial X_i}\delta X|$), δA can be written as:

$$\delta A = \sqrt{2A}\,\delta X \frac{\sqrt{\sum\limits_{i=1}^{m} T_i^3}}{\sum\limits_{i=1}^{m} T_i^2}$$

Here, δX is the spatial resolution (of the microscope), and τ is the time interval between two consecutive frames. The total number of data points is calculated as: $m = T_{max}/\tau = L^2/2A\tau$, with L being length of the reference channel. Moreover, one can write $T_i = i\tau$, which yields:

$$\delta A \cong \sqrt{2A}\,\delta X\,\sqrt{\tau}\frac{\sqrt{\frac{1}{4}m^4}}{\frac{1}{3}m^3} = \frac{3\sqrt{2}\,\sqrt{A\tau}\,A\,\delta X}{L^2} \tag{13}$$

Here, for the sake of simplicity we have assumed $m \gg 1$. Having found δA, next we need to find a relation for $\partial\eta/\partial A$ to plug into equation (12). Equation (10) for $\partial\eta/\partial A$ yields:

$$\frac{\partial \eta}{\partial A} = -\eta^2 \frac{1}{L^*}\frac{\sum\limits_{i=1}^{n} x_i t_i}{\sum\limits_{i=1}^{n} x_i^2}$$

Deriving equations for asymptotic cases of $\eta \ll 1$ and $\eta \gg 1$ and then combining the equations yields relations that can accurately reproduce the error throughout the entire parametric space. In case of $\eta \ll 1$: $x_i = \frac{A\eta}{L^*}t_i$ and $\partial\eta/\partial A = -\eta/A$. Hence:

$$E_A|_{\eta \ll 1} = \frac{1}{\eta}\left|\frac{\partial \eta}{\partial A}\delta A\right| = \frac{3\sqrt{2}\,\sqrt{A\tau}\,\delta X}{L^2} \tag{14}$$

In case of $\eta \gg 1$: $x_i^2 = 2At_i$ and $\frac{\partial \eta}{\partial A} = \frac{2}{5}\frac{L}{A}$, yielding the following relation for E_A:

$$E_A|_{\gg 1} = \frac{1}{\eta}\left|\frac{\partial \eta}{\partial A}\delta A\right| = \frac{6\sqrt{2}}{5}\frac{\sqrt{A\tau}\delta X}{LL^*}\eta \tag{15}$$

Combining equations (14) and (15) yields:

$$E_A = \sqrt{A\tau}\,\delta X(\frac{c_1}{L^2} + \frac{c_2}{LL^*}\eta) \tag{16}$$

with $c_1 = 3\sqrt{2}$, $c_2 = \frac{6\sqrt{2}}{5}$.

Spatial error (E_x): A similar approach is adopted for determining the spatial error. E_x can be expressed as:

$$E_x = \frac{1}{\eta}\left|\frac{\partial\eta}{\partial x}\,\delta x\right| \tag{17}$$

$\frac{\partial\eta}{\partial x}$ can be found from equation (10):

$$\frac{\partial\eta}{\partial x_j} = -\frac{\eta^2}{L^*}\left\{A\frac{t_j\sum_{i=1}^{n}x_i^2 - 2x_j\sum_{i=1}^{n}x_it_i}{(\sum_{i=1}^{n}x_i^2)^2} - \frac{1}{2}\frac{3x_j^2\sum_{i=1}^{n}x_i^2 - 2x_j\sum_{i=1}^{n}x_i^3}{(\sum_{i=1}^{n}x_i^2)^2}\right\}$$

Again, E_x can be analytically found for asymptotic cases of $\eta \ll 1$, and $\eta \gg 1$:

$$E_x|_{\eta\ll 1} = \sqrt{A\zeta}\,\delta x\frac{\sqrt{3}}{{L^*}^{1/2}L^{3/2}}\sqrt{\eta} \tag{18}$$

$$E_x|_{\eta\gg 1} = \frac{2\sqrt{2}}{\sqrt{3}}\sqrt{A\zeta}\,\delta x\frac{\eta}{LL^*} \tag{19}$$

with ζ being the time interval between two consecutive frames and δx being the spatial resolution of the microscope (which may or may not be the same as τ and δX). Combining the two equations above yields:

$$E_x = \sqrt{A\zeta}\,\delta x(\frac{c_3}{{L^*}^{1/2}L^{3/2}}\sqrt{\eta} + \frac{c_4}{LL^*}\eta) \tag{20}$$

with $c_3 = \sqrt{3}$, $c_4 = \frac{2\sqrt{2}}{\sqrt{3}}$.

Our full numerical solution of the error (with no estimation about η) indicated that equations (11), (16) and (20) can very accurately estimate the error. According to these relations, at small values of η, the major source of error is the term associated with c_1 which is proportional to $\frac{\sqrt{A\tau}\,\delta X}{L^2}$ and is independent of η and L^*. At large values of η, however, the terms associated with c_2 and c_4 are dominant and the error is proportional to $\frac{\sqrt{A\zeta}\delta x}{LL^*}\eta$ which grows large with increasing η, and also may be reduced by choosing a longer test channel (L^*). Numerical value of the error for $\tau = \zeta = 10$ ms (100 fps), $\delta x = \delta X = 1$ micron, L=350 micron and L^*=50 micron, for water flowing in a reference channel with h=30 nm, suggests that error at low η range is negligible (Figure 1d). At large values of η, however, error can be large and in order to reduce the error, a higher frame rate as well as a longer test channel must be considered. For example, measuring η up to 10^4 with only 20% error is possible, if the length of test channel is increased up to 2 mm and a high speed camera with a frame rate as high as 10^4 is utilized. The spatial and temporal resolutions can be further improved by utilizing techniques such as Field Effect Transistors (FET)[1] along the test/reference conduits and cross-channel current measurements using E-beam defined metal electrodes on two sides of the channel.

It is worth noting that both η and A maybe derived from a single experiment without any need to do the reference channel experiment, i.e., by introducing water from test channel side and using equation (8) along with a least square fitting method. However, further error analysis showed that results obtained from this approach are not as accurate as the two-step approach. In particular, at small values of η ($\eta \ll 1$), $x = \frac{A\eta}{L^*}t$ which suggests that in such a case only $A \times \eta$ can be found from a single experiment.

III. Decrease in channel height after anodic bonding

Although the height of nanochannels before bonding have been very accurately measured several times with AFM to ensure the consistency between different measurements, height

of the nanochannels after bonding may not be the same as before bonding. It's known that applying too large of a voltage during anodic bonding may cause a deflection equal to the height of nanochannels, in which case nanochannels collapse. This deflection is a function of applied voltage, channels' width and the thickness of oxide layer.[2] In our case, however, we observe that increasing the voltage more than a certain value first decreases the height of channels without channels collapsing, and by continuing to increase the applied voltage finally nanochannels collapse. In order to understand this phenomenon and find a bonding recipe that ensures minimal change in the channel heights we used a hybrid channel with 16.2 nm test channel. First we bonded the silicon chip with glass by applying 250 Volts at 400^oC and performed the capillary filling experiment, after oxygen plasma. Next, we applied 300 V to the same chip and re-bonded the chip (at the same temperature) and did the capillary experiment. We continued this experiment with 400 and 450 volts too. The results are presented in Figure S1. At 250 Volts, the channels are not completely bonded and a large variation in filling speed from one channel to another is observed. (Figure S1a) Imperfect bonding at this relatively low voltage gives rise to the measured values of actual resistance to be smaller than theoretical values for some channels in Figure S1c, shown by dashed ellipses. In addition complex flow pattern between channels caused irregularities in the measured x-t curves for this chip which gave rise to large fitting errors and yielded some very large resistance values, too.

As the voltage increases to 350V filling speed of different channels become consistent. (Figure S1b and S1c) By further increasing the applied voltage to 400V, the curve maintains its shape and only shifts upward (the blue and black curves in Figure S1c) which can be explained on the basis of decrease in the channel heights by 1.5 nm in case of 400V bonding. (Figure S1d) Results of 450 V bonding –obtained by another chip of exact same dimensions – does not show appreciable difference compared with 400 V bonding. (Figure S1e) Further increasing the voltage collapses the channel, and even 450V cannot be applied to the 7 nm channels. According to our experiments 350V (at 400^oC for 300 nm silicon oxide layer) is the minimum voltage that ensures all the channels are bonded and the channel heights have been minimally affected. Of course, if any change in the

channel height is going to happen, it must be subtracted from the measured thickness of the hydration layer. In the other word, 7 angstroms is the upper limit for the thickness of the hydration layer and for example compared with previsouly measured hydration layer of 5 angstroms[3] one may argue that heights of our channels might have decreased by 4 angstroms after bonding. Finally, we would like to mention that results presented in Figure S1 allows us to conclude that the hybrid channel scheme is a reliable tool to measure very small differences in the channel heights.

After the experiments the glass layer of the bonded chip was dissolved in HF and again 300 nm oxide was grown on the Si chip. Channel heights in this chip was measured using AFM and perfect agreement with initial measurement was observed. This indicated that any deformation as a result of bonding occurs to the glass and not to the silicon chip. It can be shown that glass deflection cannot be more than a few nanometers and for such a small deflection, plastic deformation is not expected. Instead, we hypothesize that conformation of the glass to the round edges of the silicon chip, as shown in Figure S2, causes a reduction in channel height. If so, creating sharp edges can reduce the extent of this problem, and thus lithography and etching steps in the fabrication become more important. This hypothesis can explain why increasing bonding voltage from 400V to 450V did not change the channel height and suggests that 400V is enough to conform the glass capping layer to the round edges of the silicon substrate. It's also probably because of this reason that the shallowest channel tested (7 nm height) yields smallest value for the thickness of the hydration layer. (4.3 angstrom) In fact, for the 7 nm channel the etching time is shortest and the edges are expected be sharper than deeper channels. Further research is required for better understanding of the post-bonding channel geometry.

IV. Discussion

A. Obtaining the correction factor C: Deviation of the speed of capillary filling in the nanochannels from theory has been subject of different works and increase in water viscosity,[4–7] surface roughness and the dynamic contact angle,[8,9] and presence of pinned

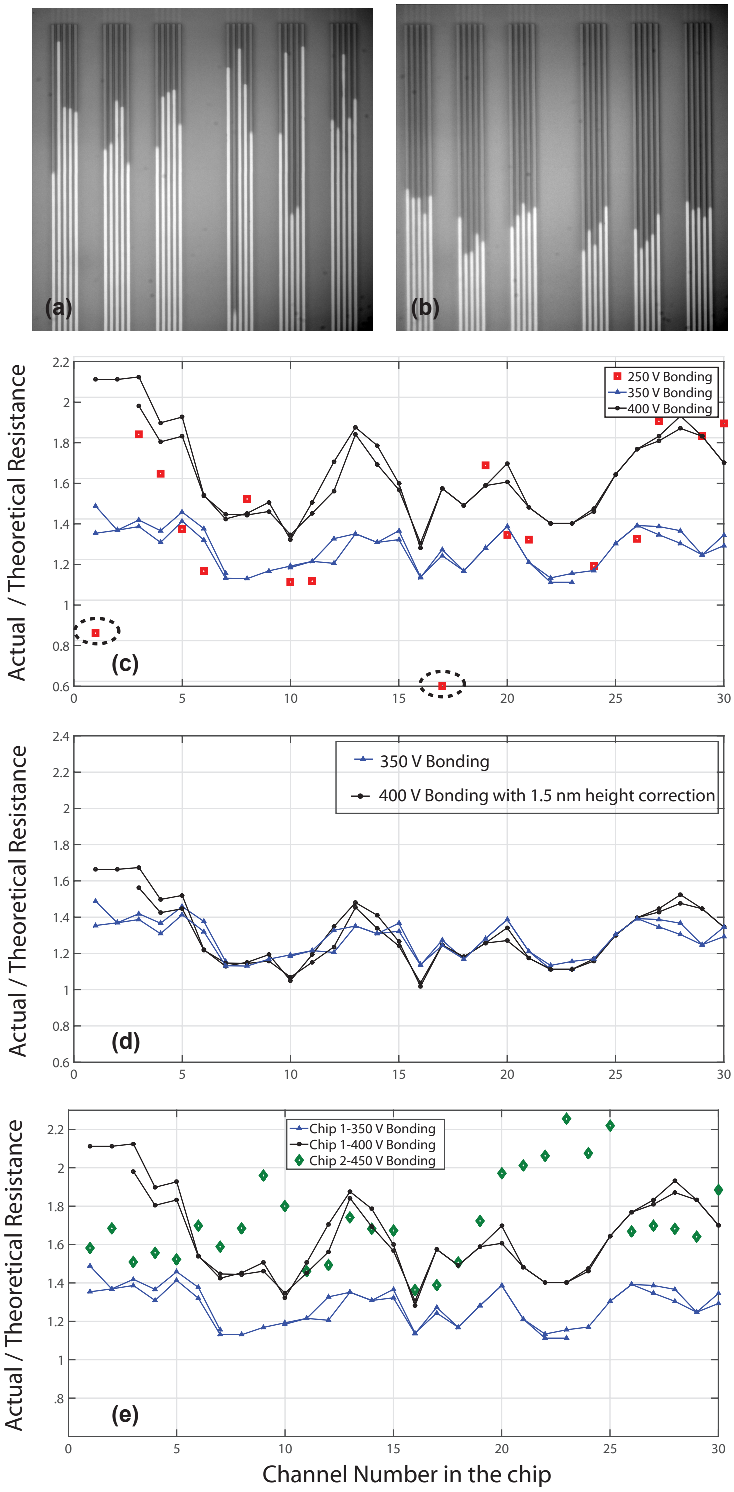

Supplementary Figure S1| Decrease in channel height after anodic bonding. (**a**) Snapshot of capillary filling in a 16.2 nm/109.5 nm hybrid channel bonded with 250V and (**b**) 350 V. 250V is not enough to form perfect bonding and thus a large variation in the capillary speed is observed. Further increasing the voltage to 350 makes all the channels consistent. (**c**) The ratio of the actual to theoretical resistance for the same chip bonded at different voltages. Two black (blue) lines are two trials under the same conditions. (**d**) Applying a 1.5nm height correction to the results obtained with 400V bonding yields very consistent results as obtained with 350V bonding. (**e**) Further increasing the voltage to 450 volts does not change the channel height.

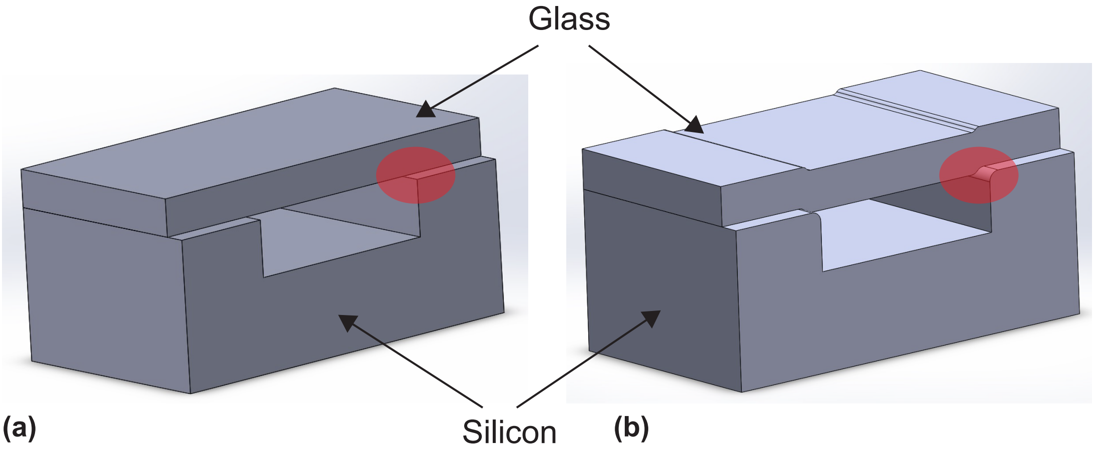

Supplementary Figure S2| Effect of round edges on post-bonding channel height. (**a**) Sharp edges prevent any change in channel height after bonding. (**b**) Capping layer conforms to the round edges (image not in scale)

bubbles or entrapment of gas inside the nanofluidic channels[10–13] are among major phenomena that have been proposed to explain it. Apparent increase in water viscosity in nanofluidic channels, known as the electroviscosity, is a phenomenon that occurs due to creation of an electro-osmosis counter flow resisting the pressure-driven flow. Dynamic contact angle is another factor that can partly explain the deviation of experiments from theory, and accounts for the fact that an advancing meniscus does not keep a constant contact angle with the walls throughout filling process, and the real average value is larger than the static contact angle. Moreover, entrapment of gas in nanofluidic channels can alter the hydraulic resistance of the channel and causes the meniscus to move slower than theory. Such deviations from theory are usually presented in the form of a correction factor defined as $C = A_{theory}/A_{actual}$, as a function of nanochannels height. In order to find the A_{actual} a curve fitting to the experimental data based on Washburn equation needs to be done. A close look at the early phase of capillary filling in the reference channel reveals that the speed of meniscus is much faster than predicted by Washburn equation (Figures S3a and 3b). Addressing this phase in capillary filling which seems to be mainly ruled by the corner flows and film flows is out of scope of this paper; nevertheless, to obtain A_{actual} one has to consider the entrance effect and hence in this work only data of menisci located in the range of 250 micron to 500 micron from the entrance of the refer-

ence channels were used to fit the theoretical curves. It's worthwhile mentioning that this phenomenon was a lot less pronounced in capillary filling of shallower reference channels with $h = 50nm$. To find the correction factor C, A_{actual} is compared with the theoretical A estimated by assuming $\Delta P = 2\sigma\cos(\theta)(\frac{1}{h}+\frac{1}{w})$, $\theta = 0$, $\mu = 1$ mPa.s, σ=0.07 N/m, w=3 micron, along with the height of the reference channel for each channel set. As already discussed, A has contributions from both resistance term as well as the pressure term. However, given the relatively large height of the reference channels and also because the electroviscosity effect cannot be more than a few percent,[4–6] negligible deviation of the hydraulic resistance from theory is expected for the reference channels (as demonstrated in Figure 4b) and the correction factor C can be mainly attributed to the capillary pressure. In fact, surface quality of the nanochannels, i.e., roughness and hydrophilicity, can be the major role players and in particular hydrophilicity of the surfaces can be slightly different from chip to chip and also may vary depending on the preparation of the chips, yielding different contact angles. In fact distorted menisci and/or menisci with different contact angles have been observed in our experiments reflecting the interplay between the capillary force and the viscous forces (Figure S3c). The contact angles shown in Figure S3c are of course the in-plane contact angles (the top view of the channel), and the capillary pressure by formation of a curved meniscus in this plane cannot be more than 3% of the total capillary pressure ($h/w \approx 3\%$); however, one can expect the same behavior in the meniscus shape to be observed along the channel height which is the dominating term.

B. Deterioration of the surface hydrophilicity, and dominance of the corner flows: Another aspect of the capillary flow at the nanoscale that we observed was the increase in the correction factor C over time. In fact, the smallest correction factor in each channel was usually achieved in the first few times a chip was tested and it increased afterwards, despite all the experiments were performed after applying oxygen plasma to the chips. Even in cases which after fabrication of a chip no experiments was performed with it and the chip was stored in a petri dish for later use, an increased correction factor C was measured the first time it was tested which clearly showed the surface

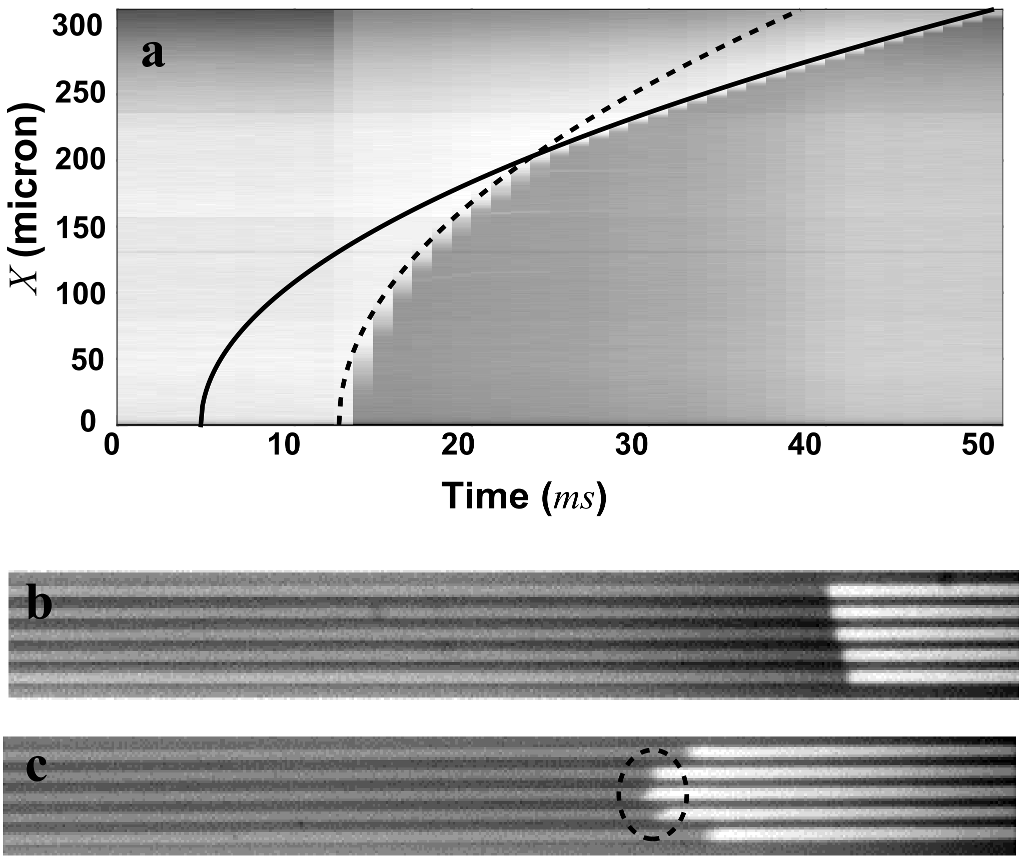

Supplementary Figure S3| Initial phase in filling and distortion of menisci. (**a**) Location of meniscus versus time in a reference channel of height h =109.5 nm. The dashed line is the best fit to the fast initial phase of filling, obtained based on the Washburn equation with the correction factor of C = 0.7. The solid line is the fit to the second phase with a correction factor of C = 1.2. (data recorded at 900 frames per second) (**b,c**) Shape of meniscus during filling can change and while in times they look normal as in **b**, they can get distorted and give rise to a reduced capillary pressure. (**c**)

quality of the channels deteriorate over time. The correction factor C for the reference channels of such a chip reached the values of up to 2 over time (Figure S4a). Therefore, for the purpose of this paper only the results of the experiments with fresh chips (right after Piranha cleaning, bonding with glass, and applying oxygen plasma) were used. However study of the old unused chips enabled us to better evaluate the performance of the hybrid nanochannels scheme, and also to get some insight about mechanism of the formation of bubbles in the nanochannels. When water is introduced from the test channel side of an old unused chip, some air pockets were trapped in several spots along the channel.[10–12] These bubbles had not been observed for most of the fresh chips of similar dimensions, or the results were discarded otherwise (Figure S4b). Repeating the experiment several times in a few consecutive days indicated that air pockets are always consistently trapped in the same spots (Figures S4c,d) and indicated that at those spots silica surface is relatively hydrophobic. On the other hand, when water was introduced from the reference channel side no air was trapped in those spots (Figure S4f), indicating that in addition to the local hydrophobicity of the surface acting as the weak points along the channel, the hydraulic resistance behind the meniscus is another factor giving rise to entrapment of air in the channel. In fact, when the meniscus reaches a hydrophobic site it is momentarily distorted. This distortion along with increase in the contact angle give rise to a reduced capillary pressure. Now if the resistance behind the meniscus is small enough to allow the reduced capillary pressure drive the water through the entire channel, no air would be trapped; otherwise, water flows from the corners and forms another meniscus downstream, leaving some air behind. Finally the air pocket forms a bubble because of the liquid pressure. (Figures S4g,h,i) Corner flows even in case of fresh chips with no hydrophobic sites can be observed. Here we experimentally observed that choice of an excessively long test channel by imposing a huge resistance to the reference channel causes the corner flows to become the dominant mode of liquid transport (Figure S5b). Since flow at the sharp corners moves quite faster than the bulk flow[14–16] and can easily fill the nanochannels–as narrow as 3 micron–with a different speed, they are considered a major problem is accurate measurement of the capillary flows. The hybrid nanochannel

design, however, gives us the latitude to eliminate or greatly reduce the corner flows by adjusting the total hydraulic resistance through right choice of test channel length (L^*) and adjusting the driving capillary pressure by right choice of the reference channel height (h).

We've been able to take advantage of the chips with deteriorated surface properties to better evaluate the hybrid nanochannel scheme and it's capability in decoupling the hydraulic resistance from the driving pressure. Using an old unused hybrid chip (a few months after bonding the chip) with h^* =28 nm experiments were performed, after applying Oxygen Plasma. By introducing water from the reference channel side the correction factor C was measured to be $C = 1.84^{+0.29}_{-0.16}$, about 50% larger than the fresh chips (Figure 4c). When water is introduced from test channel side it advances for about ~50 micron before any bubble is formed. We used this part of the data to find α for this channel height. Results showed that while the value of A in the reference channel has significantly decreased, the calculated α was consistent with the data from the fresh chips, showing an almost 10% decrease $\alpha = 1.05^{+0.16}_{-0.07}$. We hypothesize that this decrease in the hydraulic resistance can be due to formation of hydrophobic sites in the test channels similar to ones observed in the reference channels. Nonetheless, because the entire process of change in hydrophilicity of the surfaces could not be easily controlled and hence due to lack of experimental support, we could not further investigate the alteration of the hydraulic resistance of deteriorated silica channels.

C. Hybrid nanochannel scheme's advantages and disadvantages: Besides decoupling the driving capillary pressure term from the hydraulic resistance and enabling us to characterize the hydraulic resistance of nanoscale conduits, the hybrid nanochannel design offers some other advantages. For example, previous measurements of capillary flow in sub-10 nm channels[17,18] other than encompassing both deviations of the capillary force and the hydraulic resistance from theory, suffered from elastic deformation and contraction of the channel height due to the huge negative capillary pressure at the meniscus.[19–21] For a 7 nm channel the negative capillary pressure can be as high as 20 MPa,

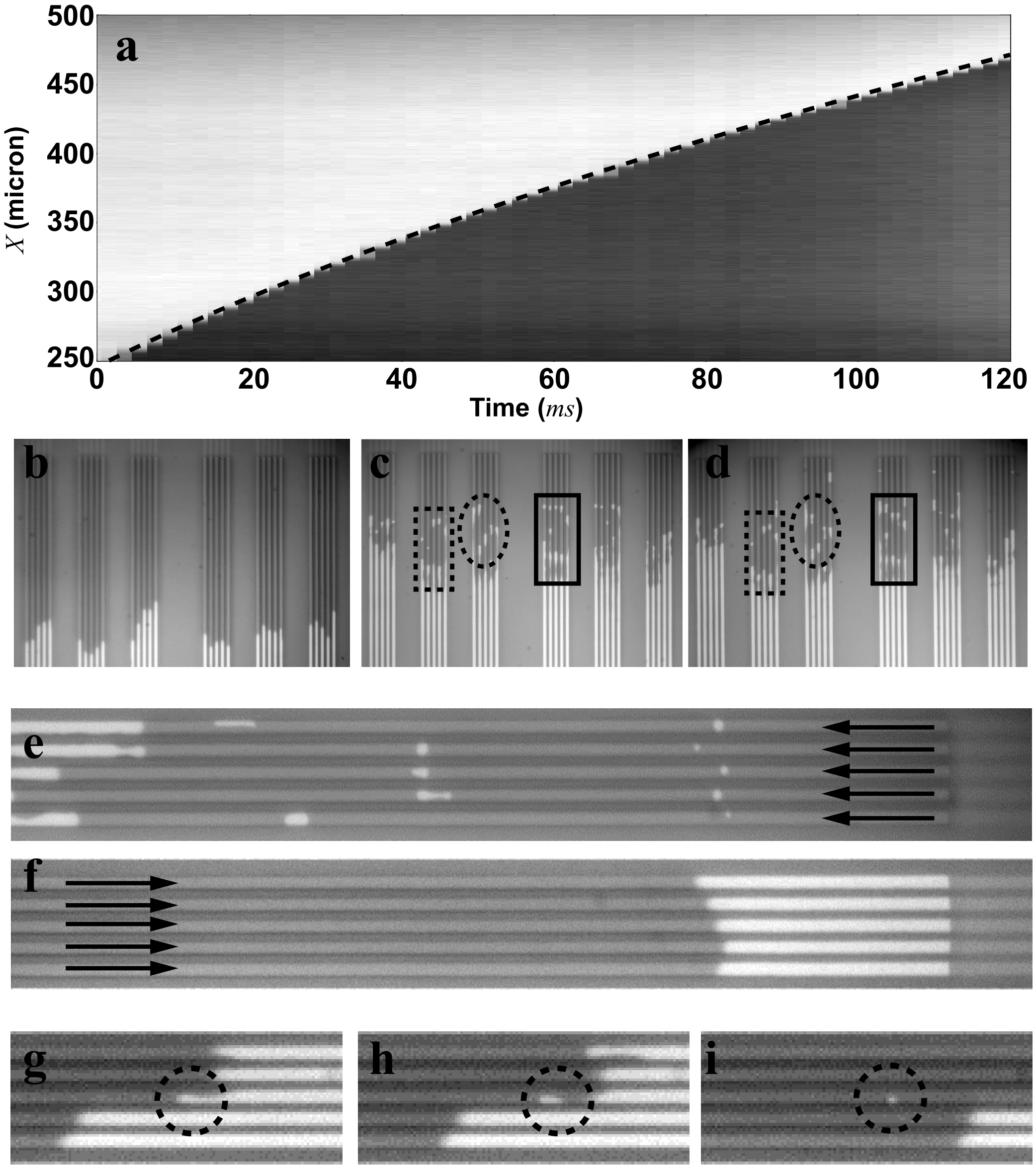

Supplementary Figure S4| Reduced capillary pressure and mechanism of formation of bubbles. (**a**) $X - t$ curve of the meniscus of a chip with deteriorated surface hydrophilicity ($C = 1.98$). (**b-d**) No trapped air or bubble is observed when water flows from test side of a fresh 28 nm hybrid channel. (**b**) For the deteriorated chip, however, bubbles are observed and they are consistently formed in the same spots. (**c** and **d**) (**e,f**) When water in introduced from the test side of a deteriorated chip (**e**), bubbles are consistently formed at several spots along the channels. But for the same chip when water in introduced from the reference side no bubble is observed. (**f**) (**g-i**) Formation of a bubble when a meniscus meets a hydrophobic site. The reduced capillary pressure at a hydrophobic site combined with a large resistance behind the meniscus make water only flow through the sharp corners, leaving bubbles behind.

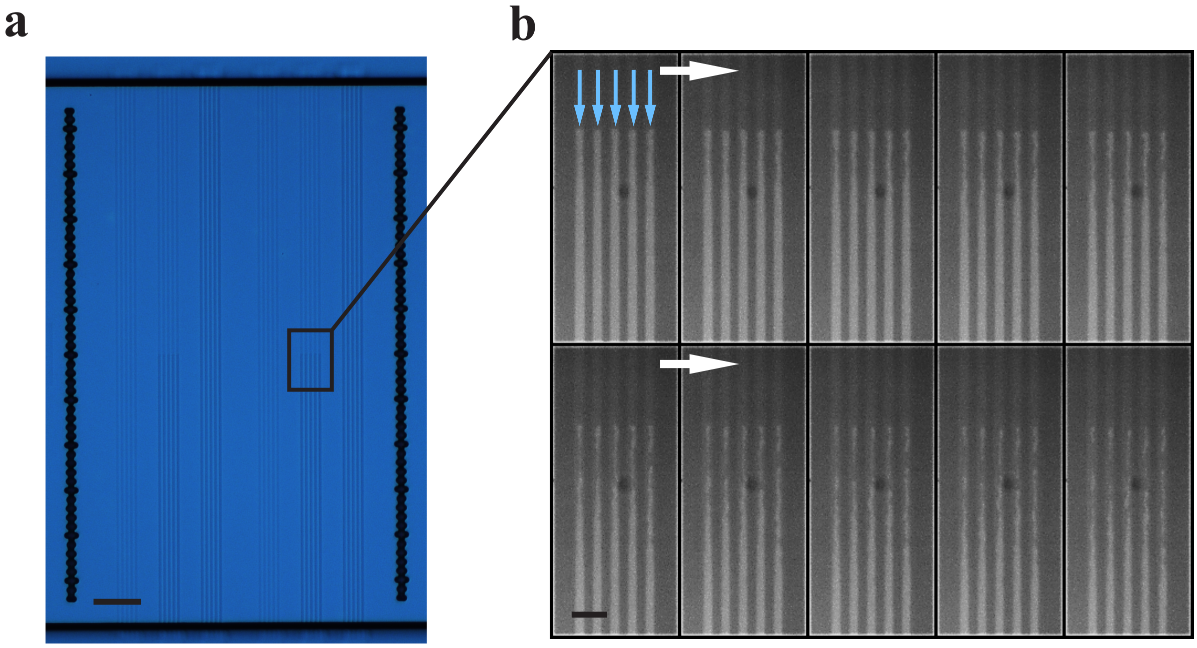

Supplementary Figure S5| Corner flows. (**a**) Microscope image of a chip consisting of hybrid nanochannels with $h^* = 18.8$ nm, $h = 39.7$ nm, and $L = L^* = 300$ micron, intended to characterize both deep and shallow nanochannels by introducing water from either sides. (**b**) 10 microscope shots of the capillary filling of five neighboring hybrid nanochannels. This design of hybrid nanochannels failed to characterize the shallow channel side, because the resistance imposed by the long test channel caused the corner flows to become the dominant mode of filling. Corner flows leave large pockets of air behind and don't allow formation of menisci. This problem was overcome by choice of shorter test channels and deeper reference channels. (scale bars in **a** and **b** are 50 and 10 micron, respectively)

which can cause a deformation in the channels at the location of meniscus, giving rise to an increased capillary pressure and also increased hydraulic resistance which is known to boost the filling rate.[19] This error, however, can be avoided using our method as data is only collected when meniscus moves in the reference channels of large height. Needless to say, in case of our measurements due to large thickness of the glass and silicon wafers (500 micron) and small width of the channels (3 micron) elastic deformation can be safely neglected, but if the nanochannels lack strong mechanical supports, this deformation can be significant. Another advantage of this method is that since only the ratio of the fluid properties in the test and reference channels determines η (and value of A is experimentally found), use of this method to a large extent eliminates the errors associated with the temperature dependence of fluid properties. This method other than characterization of the hydrophilic nanochannels can be used to characterize 1-D nanotubes and nanoporous media, even if they are hydrophobic. In fact characterization of hydrophobic conduits/nanoporous materials which don't allow for spontaneous filling can be done through their integration with a hydrophilic reference channel. Similar approach that has been explained would be used for characterization of such hydrophobic-hydrophilic hybrid channels, except that for the experiment that starts from the test channel side (hydrophobic side) we have to provide some external pressure to drive the water through the hydrophobic test channel until water enters the hydrophilic reference channel. The external pressure can be immediately removed once water enters the hydrophilic part, or it can be maintained to further drive water through the hydrophilic reference channel. In the second case, when introducing water from the hydrophilic channel side for the second measurement, the same external pressure also needs to be applied. In case of CNTs, it has been previously shown that water spontaneously fills the CNT and applying extra pressure is not needed.[1] The real challenges of CNT flow characterization include integration of CNTs in a hybrid setting and tracking the location of meniscus as a function of time. We are currently working on solving these two challenges and will report our results in another paper.

The realm of validity of the hybrid nanochannels scheme is the validity of Washburn

equation, which if for any reason violated, the method may not be applied. In addition, if the liquid of interest is non-evaporating, or cannot be removed from the hybrid channels after the first experiment, this method fails to work. Finally, rate dependence of the dynamic contact angle can introduce error to the results of this method. In our method, the velocity of meniscus can be widely different between the two experiments, i.e., the filling experiment that starts from the test channel side and another one that starts from the reference channel side, which means the driving capillary pressure can be different between the two experiments. However, in our measurements since the capillary numbers in all cases are very small ($Ca = u\mu/\sigma < 10^{-4}$ with u being the velocity), variations in $cos(\theta)$ due to different filling rates is no more than 1%,[22] consistent with previous contact angle measurements in nanochannels.[23]

References

[1] Qin, X., Yuan, Q., Zhao, Y., Xie, S. & Liu, Z. Measurement of the rate of water translocation through carbon nanotubes. *Nano Lett.* **11,** 2173–2177 (2011).

[2] Duan, C. & Majumdar, A. Anomalous ion transport in 2-nm hydrophilic nanochannels. *Nat. Nanotechnol.* **5,** 848–852 (2010).

[3] Gruener, S., Hofmann, T., Wallacher, D., Kityk, A. V. & Huber, P. Capillary rise of water in hydrophilic nanopores. *Phy. Rev. E* **79,** 067301 (2009).

[4] Mortensen, N. A. & Kristensen, A. Electroviscous effects in capillary filling of nanochannels. *Appl. Phys. Lett.* **92,** 063110 (2008).

[5] Phan, V. N., Yang, C. & Nguyen, N. T. Analysis of capillary filling in nanochannels with electroviscous effects. *Microfluid. Nanofluid.* **7,** 519–530 (2009).

[6] Wang, M., Chang, C. C. & Yang, R. J. Electroviscous effects in nanofluidic channels. *J. Chem. Phys.* **132,** 024701 (2010).

[7] Tas, N., Haneveld, J., Jansen, H., Elwenspoek, M. & Van Den Berg, A. Capillary filling speed of water in nanochannels. *Appl. Phys. Lett.* **85,** 3274–3276 (2004).

[8] Sobolev, V., Churaev, N., Velarde, M. & Zorin, Z. Surface tension and dynamic contact angle of water in thin quartz capillaries. *J. Colloid Interface Sci.* **222,** 51–54 (2000).

[9] Hamblin, M. N. *et al.* Capillary flow in sacrificially etched nanochannels. *Biomicrofluidics* **5,** 021103 (2011).

[10] Thamdrup, L. H., Persson, F., Bruus, H., Kristensen, A. & Flyvbjerg, H. Experimental investigation of bubble formation during capillary filling of sio_2 nanoslits. *Appl. Phys. Lett.* **91,** 163505–163505 (2007).

[11] van Delft, K. M. *et al.* Micromachined fabry-perot interferometer with embedded nanochannels for nanoscale fluid dynamics. *Nano Lett.* **7,** 345–350 (2007).

[12] Chauvet, F., Geoffroy, S., Hamoumi, A., Prat, M. & Joseph, P. Roles of gas in capillary filling of nanoslits. *Soft Matter* **8,** 10738–10749 (2012).

[13] Han, A., Mondin, G., Hegelbach, N. G., de Rooij, N. F. & Staufer, U. Filling kinetics of liquids in nanochannels as narrow as 27 nm by capillary force. *J. Colloid Interface Sci.* **293,** 151–157 (2006).

[14] Ransohoff, T. & Radke, C. Laminar flow of a wetting liquid along the corners of a predominantly gas-occupied noncircular pore. *J. Colloid Interface Sci.* **121,** 392–401 (1988).

[15] Weislogel, M. M. & Lichter, S. Capillary flow in an interior corner. *J. Fluid Mech.* **373,** 349–378 (1998).

[16] Dong, M. & Chatzis, I. The imbibition and flow of a wetting liquid along the corners of a square capillary tube. *J. Colloid Interface Sci.* **172,** 278–288 (1995).

[17] Haneveld, J., Tas, N. R., Brunets, N., Jansen, H. V. & Elwenspoek, M. Capillary filling of sub-10nm nanochannels. *J. Appl. Phys.* **104,** 014309 (2008).

[18] Oh, J. M., Faez, T., de Beer, S. & Mugele, F. Capillarity-driven dynamics of water–alcohol mixtures in nanofluidic channels. *Microfluid. Nanofluid.* **9,** 123–129, (2010).

[19] Van Honschoten, J., Escalante, M., Tas, N., Jansen, H. & Elwenspoek, M. Elastocapillary filling of deformable nanochannels. *J. Appl. Phys.* **101,** 094310 (2007).

[20] Tas, N. R., Mela, P., Kramer, T., Berenschot, J. & van den Berg, A. Capillarity induced negative pressure of water plugs in nanochannels. *Nano Lett.* **3,** 1537–1540 (2003).

[21] Van Honschoten, J., Escalante, M., Tas, N. & Elwenspoek, M. Formation of liquid menisci in flexible nanochannels. *J. Colloid Interface Sci.* **329,** 133–139 (2009).

[22] Ralston, J., Popescu, M. & Sedev, R. Dynamics of wetting from an experimental point of view. *Annu. Rev. Mater. Res.* **38,** 23–43 (2008).

[23] Li, L., Kazoe, Y., Mawatari, K., Sugii, Y. & Kitamori, T. Viscosity and wetting property of water confined in extended nanospace simultaneously measured from highly-pressurized meniscus motion. *J. Phys. Chem. Lett.* **3,** 2447–2452 (2012).
